# DOES LOSS OF A LOVED ONE TO COVID-19 MODERATE THE ASSOCIATION OF LONELINESS AND MENTAL HEALTH AMONG OLDER PUERTO RICANS?

**DOI:** 10.1093/geroni/igac059.622

**Published:** 2022-12-20

**Authors:** Matthew Morgan, Seon Kim, Todd Becker, Thomas Buckley

**Affiliations:** Virginia Commonwealth University, Richmond, Virginia, United States; Virginia commonwealth university, Richmond, Virginia, United States; University of Maryland, Baltimore, Baltimore, Maryland, United States; University of Pittsburgh, Pittsburgh, Pennsylvania, United States

## Abstract

Loneliness is a well-established risk factor for poor mental health. During COVID-19, loneliness and mental health have been exacerbated by widespread disease-related mortality, suggesting that loss of a loved one may influence this relationship. Using data from 187 older adults in Puerto Rico, we assessed the association between loneliness and mental health and the potential moderating role of loss. Moderated multivariable linear regression results indicated that loneliness was significantly, positively associated with mental health (B = 1.58, p < .001). Although loss due to COVID-19 was not significantly associated with mental health (p = .473), it did moderate the relationship between mental health and loneliness (B = −1.00, p = .048). The lack of significant association of loss and mental health contrasts with previous research on COVID-19 and warrants further investigation. Nevertheless, the statistically significant interaction suggests that grief should be considered when assessing individual and community support during the pandemic.

